# Lipid-dependent sequential allosteric activation of heat-sensing TRPV1 channels by anchor-stereoselective “hot” vanilloid compounds and analogs

**DOI:** 10.1016/j.bbrep.2021.101109

**Published:** 2021-09-02

**Authors:** Guangyu Wang

**Affiliations:** a Department of Physiology and Membrane Biology, University of California School of Medicine, Davis, CA, USA; b Institute of Biophysical Medico-chemistry, Reno, NV, USA

**Keywords:** Sequential cooperativity, Active site stereoselectivity, Dominant steady-state ligand binding, Recessive transient ligand binding, Lipid-ligand interaction, Hydrogen bonding network, Ion channel

## Abstract

Both a silent resident phosphatidylinositol lipid and a “hot” vanilloid agonist capsaicin or resiniferatoxin have been shown to share the same inter-subunit binding pocket between a voltage sensor like domain and a pore domain in TRPV1. However, how the vanilloid competes off the resident lipid for allosteric TRPV1 activation is unknown. Here, the *in silico* research suggested that anchor-stereoselective sequential cooperativity between an initial recessive transient silent weak ligand binding site and a subsequent dominant steady-state strong ligand binding site in the vanilloid pocket may facilitate the lipid release for allosteric activation of TRPV1 by vanilloids or analogs upon non-covalent interactions. Thus, the resident lipid may play a critical role in allosteric activation of TRPV1 by vanilloid compounds and analogs.

## Highlights

• Four active vanilloid binding pockets as revealed by the cryo-EM structure of TRPV1 have no cooperativity between subunits.
• Allosteric activation of TRPV1 by vanilloid compounds and analogs is lipid-dependent and anchor-stereoselective.
• The resident and occluded lipid must be competed off for allosteric activation of TRPV1 by vanilloid compounds and analogs.
• The first ligand binding is needed to release the resident lipid for the second ligand binding in the vanilloid pocket.
• A lipid-free anchor facilitates the vanilloid ligand binding to remove the resident lipid from the active site in TRPV1.
• Site accessibility controls sequential cooperative interactions of TRPV1 with vanilloids or analogs to open the channel.
• The anchor stereoselectivity depends on the formation of a vanilloid bridge between two separated residues at the active site.
• Different anchor stereoselectivities produce diverse recessive transient reaction intermediates or steps for TRPV1 opening.
• Membrane hyperpolarization and depolarization may stabilize and loosen the resident lipid for TRPV1 gating, respectively.
• Phospholipids at N- and C- terminal domains may affect the cooperativity or the potency of the vanilloid ligands.

## 1 Introduction

The kinetics of a given biochemical or physiological reaction or process often reveal very powerful insight into reaction mechanisms. For example, the rate of an enzyme-catalyzed reaction or a carrier-mediated membrane transport process as a function of the substrate or ligand concentration can provide valuable clues about the nature of substrate or ligand binding to the enzyme or the transporter. The rate changes in a hyperbolic or sigmoidal manner as the substrate or ligand concentration increases can be described by a Michaelis-Menten equation or a Hill equation. A fundamental parameter of the Hill equation is the Hill coefficient (h). When it is 1, the Hill equation becomes the Michaelis-Menten equation (Table 1).

**Table 1:** Comparison of the Hill coefficient (h) of vanilloid or analog dose responses of TRPV1 channels.

Ligand	Ion channel source	V_h_, mV	h^a^	Ref
resiniferatoxin	rTRPV1	−60	1.95	[2]
capsaicin	rTRPV1	−60	2.08	[2]
capsaicin	mTRPV1	+80	1.82	[5]
capsaicin	Y512F/mTRPV1	+80	1.31	[5]
capsaicin	S513A/mTRPV1	+80	1.23	[5]
capsaicin	F544A/mTRPV1	+80	1.13	[5]
capsaicin	R558L/mTRPV1	+80	1.19	[5]
capsaicin	I662A/mTRPV1	+80	0.92	[5]
capsaicin	A666L/mTRPV1	+80	1.20	[5]
capsaicin	T671V/mTRPV1	+80	0.98	[5]
capsaicin	T671S/mTRPV1	+80	1.28	[5]
capsaicin	CFTR	±100	1.92	[31]
peperine	mTRPV1	0	2.20	[24]
peperine	T551V/mTRPV1	0	1.3	[24]
6-Shogaol	mTRPV1	+80	1.10	[25]
6-Gingerol	mTRPV1	+80	1.70	[25]
6-Zingerone	mTRPV1	+80	0.90	[25]
capsazerpine	mTRPV1	+80	1.38	[5]
Curcumin	mTRPV1	−60	0.88	[28]
Curcumin	mTRPV1	−60	0.89	[30]
Naicin	hTRPV1	+80	2.87	[26]
Histamine	mTRPV1		3.82	[36]

a h values were based on the non-linear curve fitting of the Hill equation, I=I_max_*[L]^h^/(K_1/2_^h^ +[L]^h^)_,_ where, I_max_ is the maximum effect; L is the ligand; K_1/2_ is the ligand concentration that produces a 50% maximal response and h is the Hill coefficient.

In most cases, given that the rate of a large amount of enzyme-catalyzed reactions or carrier-mediated transport processes across biological membranes exhibit a sigmoidal behavior, the Hill coefficient h is commonly employed to indicate the cooperativity of two or more substrate or ligand binding sites. The h value greater than 1 is usually indicative of two of more cooperative substrate or ligand binding sites in the allosteric protein. In this case, the binding of one substrate or ligand to one subunit promotes the binding of another substrate or ligand to another subunit in a sequential cooperative mechanism. The higher the Hill coefficient is, the stronger the substrate or ligand potency becomes. For example, the quinoprotein glycine oxidase from the marine bacterium *Pseudoalteromonas luteoviolacea* (PlGoxA) employs a protein-derived cysteine tryptophylquinone (CTQ) cofactor to catalyze conversion of glycine to glyoxylate and ammonia. This homotetrameric enzyme exhibits strong cooperativity toward glycine binding. When the F316A or Y766F mutation at the inter-subunit active-site weakens the glycine binding cooperativity, both the enzyme catalysis potency and the glycine binding potency are also decreased [1].

**Fig. 1:**
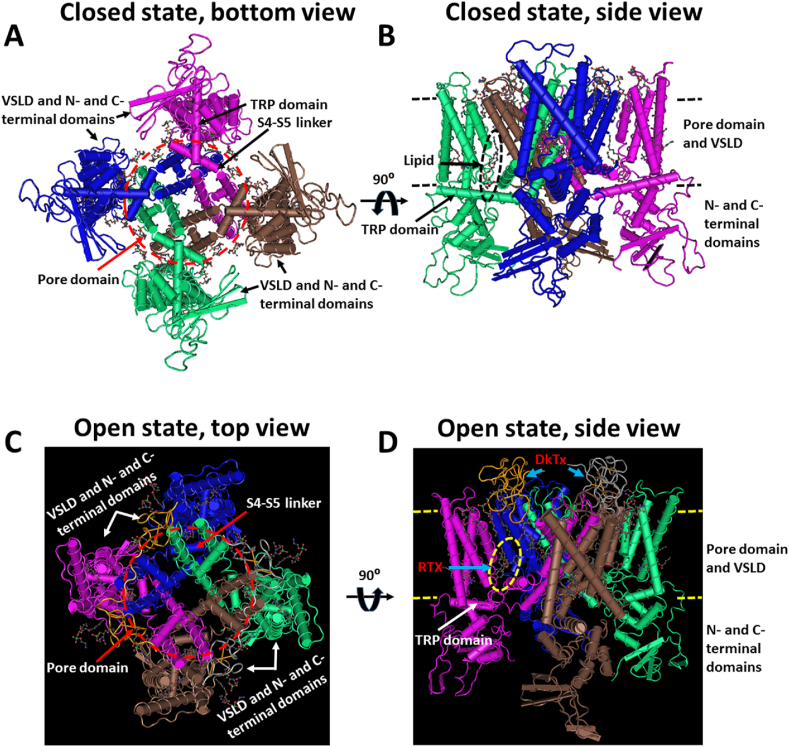
**The overall cryo-EM structure of TRPV1**. The bottom (**A**) and side (**B**) views of the homotetrameric arrangements of channel subunits are shown in the cryo-electron microscopy (EM) structures of TRPV1 with four resident phosphatidylinositol lipids bound for the closed state (PDB ID: 5IRZ) [3]. In contrast, the top (**C**) and side (**D**) views of the homotetrameric arrangements of channel subunits are indicated in the cryo-EM structures of TRPV1 with two spider toxin DkTx and four resiniferatoxin (RTX) bound for the open state (PDB ID: 5IRX) [4]. Four subunits with different colors form a tetramer. The positions of the lipid bilayer are shown with black (**B**) and yellow (**D**) dotted lines. The extracellular side starts from the top line while the cytoplasmic side ends in the bottom line. Four transmembrane helices S1–S4 serve as a voltage sensor-like domain (VSLD) while other two transmembrane helices S5–S6 function as a pore domain (red circled). The pore domain is circled by the VSLD in a domain-swapped arrangement via the S4–S5 linker (**A, C**). The cation conductance pathway centers in the pore domain and an intervening pore loop region (**A, C**). A wide outer vestibule with a short flexible selectivity filter is cradled by the VSLD domain. Channel opening is controlled by downward movement of the pore helix between S5 and S6 and coupled dilation of both a selectivity filter and a hydrophobic constriction at the lower S6 gate. The conserved TRP domain links not only the S6 bundle crossing gate but also interacts with the S4–S5 linker promoting allosteric coupling between channel domains. The characteristic ankyrin repeats within the cytoplasmic N terminus facilitate channel assembly by tethering cytoplasmic N- and C-terminal domains [3,4]. A yellow circle is one of four RTX binding sites (**D**), which share the same location with four resident lipid binding sites, one of which is indicated by a black circle (**B**). The RTX or lipid binding pocket is formed by the S3 and S4 transmembrane segments, the S5 and S6 segments from a neighboring subunit, and the S4–S5 linker. (For interpretation of the references to color in this figure legend, the reader is referred to the Web version of this article.)

In contrast, transient receptor potential (TRP) vanilloid-1 (TRPV1) channel is also homotetrameric. Its allosteric activation by a vanilloid agonist capsaicin or resiniferatoxin (RTX) also exhibits a cooperative dose response with a Hill coefficient greater than 1. Since the cryo-electron microscopy (cryo-EM) structures of TRPV1 with bound capsaicin or RTX demonstrated that only one capsaicin or RTX molecule binds to an active pocket between a voltage sensor like domain and a pore domain in homotetrameric TRPV1 (Table 1; Fig. 1) [[2], [3], [4]], at least two vanilloid agonists should have binding cooperativity through inter-subunit communication. However, when the tail length (CH_x_)_n_ of capsaicin decreases from 11 to 0, the Hill coefficient lowers from 2 to 1 [5]. Thus, the four dominant steady-state active vanilloid binding pockets as revealed by the cryo-EM structure of TRPV1 have no cooperativity between subunits. In this case, the remaining question is how a vanilloid agonist activates TRPV1 in a cooperative mechanism.

This review examines structural aspects of how the resident lipid modulates allosteric TRPV1 channel activation by “hot” vanilloid agonists from pepper and ginger and their analogs. The vanilloid agonists include capsaicin, resiniferatoxin, 6-Gingerol, 6-Zingerone, and 6-Shogaol. The vanilloid analogs cover peperine and niacin. The relationships between the protein structure of TRPV1 and the chemical structures of those ligands were summarized by the *in silico* analysis. In addition, the relationships between the resident lipid and TRPV1 vanilloid agonists and analogs are discussed. How capsazepine or curcumin competes off vanilloid agonists to inhibit channel activity is also discussed. Therefore, this review is informative to understand pharmacological data of vanilloid effects on TRPV1 in the future study.

Given that the cryo-electron microscopy (EM) structure has not captured the ligand binding in the presence of the resident lipid, an initial recessive transient silent ligand binding site may play a critical role in allosteric activation of TRPV1 by vanilloid agonists. The *in silico* research suggested that a lipid-dependent cooperativity between an initial recessive transient silent ligand binding site and a subsequent dominant steady-state active ligand binding site in the vanilloid pocket may favor the release of the resident and occluded lipid in the vanilloid pocket. This lipid-dependent cooperativity may also promote allosteric activation of TRPV1 channels by the vanilloid or its analog in a sequential concerted but anchor-stereoselective mechanism. Further *in silico* analyses indicate which anchor in TRPV1 is first used for the agonist to bind to release the resident and occluded lipid, how many reaction intermediates or steps are needed for final activation of TRPV1, and how a membrane potential exerts effects on the allosteric activation of TRPV1. In this regard, this review may also update our understanding of the lipid-dependent stereoselective interplay between a ligand and an allosteric membrane protein receptor.

## 2 TRPV1 is a receptor of “hot” vanilloid compounds

TRPV1 is a non-selective cation channel located in the plasma membrane of nociceptive sensory neurons of the dorsal root ganglia (DRG) and the trigeminal ganglia (TG). Upon channel opening, an influx of Na^+^ causes the neuron to depolarize while an influx of Ca^2+^ transmits signals similar to those that would be transmitted if the innervated tissue were being burned or damaged. TRPV1 functions as a polymodal nociceptor in response to extensive physical and chemical stimuli. It can be activated not only by heat and membrane depolarization but also acidic PH and divalent cations and spider toxins and vanilloids [2,[6], [7], [8], [9]]. In most *in vitro* studies, the vanilloids such as capsaicin and RTX have been shown to act on ion channels at a concentration range from 0.3 nM to 10 μM [2,7].

**Fig. 2:**
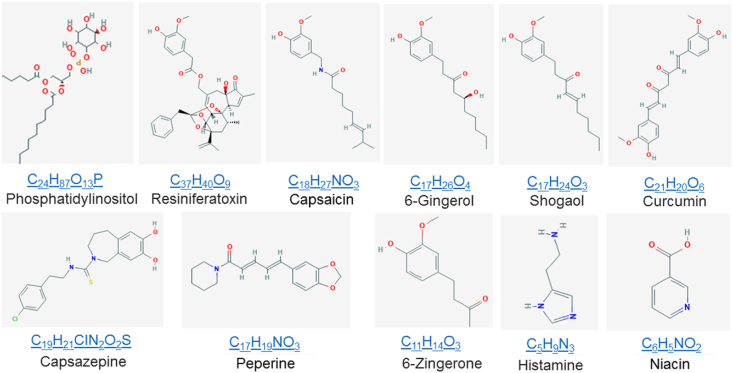
**Structural comparison of vanilloid compounds and analogs and phosphoinositol**. All the vanilloid compounds have a vanillyl group (methoxyphenol) as a head while the middle C=O group or an amide bond group as a neck and the hydrophobic carbon chain as a tail. Resiniferatoxin (RTX) and 6-Gingerol and shogaol and capsaicin have a long tail; 6-Zingerone has a very short tail; Peperine and curcumin have a long neck; Curcumin has a large tail. Curcumin and capsazepine with a different neck and a polar tail serve as an antagonist while others are agonists for TRPV1. Peperine and histamine and niacin are vanilloid analogs. Phosphoinositol is a control with a hydrophilic head and a hydrophobic tail and silent in the vanilloid pocket.

The most notable vanilloid is pungent capsaicin from ‘hot’ spicy peppers or *Capsicum annuum* (Fig. 2). The binding of capsaicin to the heat-gated TRPV1 channel evokes a burning sensation [2,6]. Capsaicin is also a neuropeptide-releasing agent selective for primary sensory peripheral neurons. Its ability to desensitize TRPV1 causes it to serve as a non-narcotic topical analgesic in many formulations of cream, liquid, and patch preparations of various strengths to relief peripheral nerve pain.

RTX from *Euphorbia resinifera* is an ultra-potent TRPV1 agonist with ~500-fold higher potency for TRPV1 than capsaicin (Fig. 2). It is rather toxic and can inflict chemical burns in tiny quantities by activating TRPV1 in a subpopulation of primary afferent sensory neurons responsible for the perception of pain [10,11]. This stimulation is followed by desensitization and analgesia, partly because the nerve endings die from calcium overload.

Capsazepine was first reported as a capsaicin receptor antagonist (Fig. 2). It blocks the activation of TRPV1 cation channels by capsaicin to relief the painful sensation of heat. The IC_50_ is ranged from nanomolar to low micromolar. Although it can also activate the noxious chemical sensor TRPA1 [12], or inhibit the cold-activated TRPM8 channel [13], voltage-activated calcium channels [14], and nicotinic acetylcholine receptors [15], capsazepine is mainly used as a tool to study the TRPV1 channel.

## 3 The overall cryo-EM structure of TRPV1

The 3D cryo-EM structures have confirmed the overall similarity of TRPV1 to other tetrameric cation channels (Fig. 1) [[3], [4], [16]]. For example, four transmembrane helices S1–S4 act as a voltage sensor-like domain (VSLD) while other two transmembrane helices S5–S6 function as a pore domain. The pore domain is circled by the VSLD in a domain-swapped arrangement. The cation conductance pathway centers in the pore domain and an intervening pore loop region. A wide outer vestibule with a short flexible selectivity filter is cradled by S1–S4 domains. Channel opening is controlled by downward movement of the pore helix between S5 and S6 and coupled dilation of both a selectivity filter and a hydrophobic constriction at a lower S6 gate. The conserved TRP domain not only links the S6-gate bundle but also interacts with the S4–S5 linker promoting allosteric coupling between channel domains. The characteristic ankyrin repeats within the cytoplasmic N terminus facilitate channel assembly by tethering cytoplasmic N- and C-terminal domains [[3], [4], [16]].

A spider double-knot toxin or vanillotoxin (DkTx) can be used to stably trap TRPPV1 in its fully open state. DkTx is a bivalent tarantula peptide and has a short (7 amino acid) linker to join two nearly identical inhibitor cysteine knot (ICK) motifs. When two DkTx molecules bind to residues within the S5–P–S6 pore region of one TRPV1 tetramer, the antiparallel linker connects the ICK knots and three canonical disulfide bonds are formed so that two DkTx molecules ‘staple’ TRPV1 together in its activated state [4].

When lipid nanodisc technology was used to ascertain the structure of the TRPV1 ion channel in a native bilayer environment, it was found that specific phospholipid interactions enhance binding of DkTx to TRPV1 through formation of a tripartite complex. What is more, phosphatidylinositol lipids share the same binding site with capsaicin and other vanilloid ligands. The lipid's branched acyl chains extend upward between S4 of one subunit and S5 and S6 of an adjacent subunit, within a hydrophobic cleft facing the lipid bilayer. Thus, bioactive lipids must be released for chemical stimuli to elicit critical allosteric channel activation [4].

## 4 The vanilloid bridge between E571 on the S4–S5 linker and T551 anchor against S4 of mouse TRPV1 may be stimulatory for capsaicin and its analogs

**Fig. 3:**
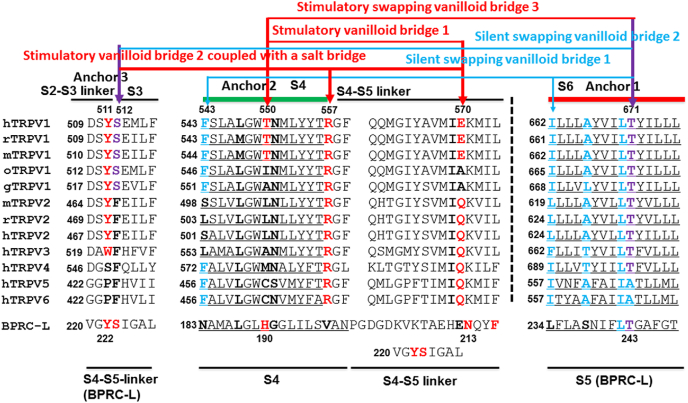
**Sequence alignment of TRPV channel family members. Residues discussed in the text are bolded and colored**. Numbers above and below the sequences refer to the human TRPV1 (hTRPV1) channel and the purple bacterial reaction center (PBRC) from *Rhodobacter sphaeroides*, respectively. The segment of PBRC from V220 to L227 can be aligned with either the S2–S3 linker/S3 or the S4–S5 linker of TRPV1. The critical residues for stimulatory non-swapping vanilloid bridges 1 and 2 and swapping bridge 3 are indicated in red while those for the silent swapping vanilloid bridges 1 and 2 are shown in blue. The residues shared by both silent and stimulatory vanilloid bridges are indicated in purple. Three anchors are proposed for a vanilloid compound to bind during the formation of the stimulatory and silent vanilloid bridges. The anchor T550 (T551 in mTRPV1) in S4 is arrowed in red while the anchor S512 (S513 in mTRPV1) in S3 and the anchor T671 (T670 in rTRPV1) in S6 are arrowed in purple. The black dash line separates two subunits of TRPV channels. (For interpretation of the references to color in this figure legend, the reader is referred to the Web version of this article.)

Since identification and cloning of capsaicin receptor TRPV1 in 1997 [2], chimeric studies of capsaicin-sensitive human TRPV1 (hTRPV1) and capsaicin-insensitive chicken (gTRPV1) or rat TRPV2 (rTRPV2) indicated that the S2–S4 region is involved in capsaicin activation. The subsequent sequence alignment of rTRPV1 and rTRPV2 and gTRPV1 and mutagenesis studies based on a capsaicin-bound bacterial photosynthetic reaction center (BPRC) further narrowed down Y511 and S512 in the cytosolic S2–S3 linker as capsaicin-sensitive residues (Fig. 3). Since the previous X-ray crystallographic structure indicated that the vanillyl group of capsaicin binds at the Q_B_ pocket and stacks with F216, Y511 was also proposed to interact with the vanillyl group of capsaicin [7,17]. Later other chimeric and mutagenesis studies on rTRPV1 and oTRPV1 identified M547 and T550 as additional capsaicin-sensitive residues [[18], [19], [20]]. In that case, the vanillyl group of capsaicin was proposed to interact with T550 against the nearby W549 while the hydrophobic tail faces Y511 [19].

A decade later the cryo-EM structure of rTRPV1, which was combined by the vanilloid agonist RTX or capsaicin in the presence of vanillotoxin (called double-knot toxin, DkTx), confirmed that the RTX or capsaicin pocket is formed by S512 on S3, Y511 on the S2–S3 linker, M547 and T550 on S4 and E570 on the S4–S5 linker from the same subunit, and L669 on S6 of a neighboring subunit [3]. Although the interaction of RTX or capsaicin with both S6 and the S4–S5 linker was proposed to affect channel gating upon reorientation of Y511, the mechanistic insight into the interplay between TRPV1 and RTX or capsaicin is still missing at the atomic level, in part because of the weak signal observed with capsaicin and the presence of electron density in this vanilloid pocket in the apo rTRPV1 structure [3]. In this regard, based on molecular docking of capsaicin with different tail lengths and systematic site-specific mutagenesis studies from Y511 to L674 of mouse TRPV1 (mTRPV1), it was proposed that capsaicin takes “tail-up, head-down” configurations. In this model, in addition to extensive but nonspecific van der Waals interactions of the aliphatic tail with residues lining the binding pocket, T551 anchors the amide group for the vanillyl head group to H-bond with the side chain of E571 so as to uplift the S4–S5 linker away from the pore for channel opening [5]. Although the subsequent cryo-EM structural model of rTRPV1 with resiniferatoxin or the competitive vanilloid antagonist capsazepine bound in lipid nanodiscs supported this model, the capsaicin-bound cryo-EM structural model with high electron density in a native state is still missing [4].

## 5 The vanilloid cooperativity is residue site-dependent

When the ligand binding at two or more active sites is positively cooperative, a Hill coefficient of a ligand dose response is indicative of the minimum number of individual ligand molecules required for significant allosteric activation of multimeric proteins. That means that the affinity for ligand binding to an open site on one subunit is enhanced by binding of a ligand to another subunit. Thus, for this inter-subunit sequential allosteric ligand binding, the Hill coefficient will change with a variation of ligand potency. For example, two cyclic guanosine monophosphate GMP (cGMP), once linked with a polymer with an optimal length, are up to a thousand times more potent than cGMP alone in activating cyclic-nucleotide-gated (CNG) channels in vertebrate photoreceptors and olfactory neurons, and the resultant Hill coefficient is decreased from two to one [21]. Thus, two sequential cooperative cGMP ligands are necessary for allosteric activation of CNG channels. In contrast, if the ligand binding sites are not cooperative, the Hill coefficient should not change when the potency is altered. For example, a single engineered Zn^2+^ site in cystic fibrosis transmembrane conductance regulator (CFTR) always keeps a constant Hill coefficient of a Zn^2+^ dose response although the site-mutation at the metal site decreases the Zn^2+^ potency [22].

**Fig. 4:**
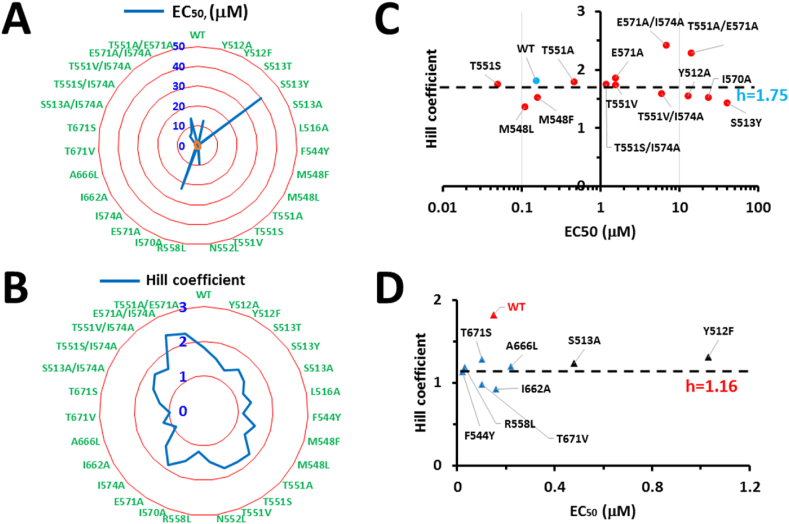
**The residue site-dependent Hill coefficients and potency of a capsaicin dose response for mTRPV1**. **A**, Radar scanning for the site mutation-induced changes in the capsaicin potency (EC_50_) of mTRPV1. **B,** Radar scanning for the site mutation-induced changes in the Hill coefficient of a capsaicin dose response of mTRPV1. Upon radar scanning, the residues in the lipid pocket can be divided into two populations as shown in panels **C** and **D** on the basis of the effects of their site mutations on the changes in the Hill coefficient and the capsaicin potency. **C,** The residues for the non-swapping vanilloid bridge via the side chains between T551 on S4 and E571 on the S4–S5 linker are not cooperative. Their site-directed mutations dramatically change the capsaicin potency (EC_50_) of mTRPV1 but do not significantly alter the Hill coefficient of a capsaicin dose response. WT is a control colored in blue and other mutants are colored in red. **D,** The lipid-free residues for the swapping vanilloid bridge via the side chains between T671 on S6 and F544 on S4 are cooperative. Their site-directed mutations significantly decrease the Hill coefficient of a capsaicin dose response but do not dramatically alter the capsaicin potency (EC_50_) of mTRPV1. WT is a control colored in red and other mutants are colored in blue. Data were produced from the published article [5]. (For interpretation of the references to color in this figure legend, the reader is referred to the Web version of this article.)

In the case of homotetrameric TRPV1 with four capsaicin binding pockets, although Y512, S513, M548, T551, E571 of mTRPV1 are capsaicin-sensitive residues, their mutation-induced changes in the capsaicin potency and the Hill coefficient are independent of each other. Therefore, these four steady-state capsaicin binding pockets are energetically independent and equivalent and have no cooperativity (Fig. 1, Fig. 2, Fig. 3, Fig. 4). This notion is further supported by the finding that the Hill coefficient of a capsaicin dose response is decreased once the tail length of capsaicin is shortened (Fig. 4). More importantly, both shogaol and 6-Zingerone bind to the same capsaicin pocket for activation of TRPV1 but only exhibit a non-cooperative dose response with a low Hill coefficient (Table 1). In that regard, the higher Hill coefficient of a capsaicin dose response may not be indicative of the minimum number of individual capsaicin molecules required for the significant allosteric activation of TRPV1 channels. Since the sarcoplasmic reticulum Ca^2+^-ATPase reactions show that a high Hill coefficient of a substrate dose response may also suggest multi-step transient cooperative reaction intermediates at one active binding pocket in the monomeric protein [23], it is fitting to ask if there may exist multi-step transient cooperative reaction intermediates at one active vanilloid binding pocket of TRPV1.

## 6 Allosteric activation of TRPV1 by capsaicin is lipid-dependent

A phosphatidylinositol lipid was observed within the vanilloid binding pocket of the apo rTRPV1 (Fig. 1, Fig. 6). The inositol ring above the TRP domain is anchored against S3 and the elbow of the S4–S5 linker. The lipid is further stabilized by ionic interactions of negatively charged phosphate or hydroxyl moieties on inositol ring with positive and negative residues from S4 and S4–S5 linker, respectively. They include, but not limited to, polar interactions between the side chain of R557 on S4 and the OH- group of the phosphate on position 1, or between the side chain of E570 in the S4–S5 linker and a OH- group on position 6 of the inositol ring, and electrostatic interactions between phosphate groups at positions 3, 4 and/or 5 of the inositol ring and the side chain of R409 in a cytoplasmic N-terminal segment preceding S1 or the side chains of K571 and R575 within the S4–S5 linker [4].

The cryo-EM structures of rTRPV1 demonstrated that capsaicin and RTX and capsazepine share the same binding pocket with a silent resident phosphatidylinositol lipid (Fig. 1). This pocket is half-surrounded by S3, S4, a S2–S3 linker and a S4–S5 linker from one subunit, and S5 and S6 from the adjacent subunit [3,4]. In this case, vanilloid compounds must compete off the silent resident lipid from the open side of the pocket to activate TRPV1 channels. Since R558 on S4 is critical for the lipid head binding but the R558L mutation of mTRPV1 lowers the Hill coefficient of a capsaicin dose response from 1.82 to 1.19 (Fig. 1, Fig. 2, Fig. 3, Fig. 4, Table 1) [5], allosteric activation of TRPV1 by capsaicin is lipid-dependent.

## 7 Allosteric activation of TRPV1 by vanilloid agonists is anchor-stereoselective

**Fig. 5:**
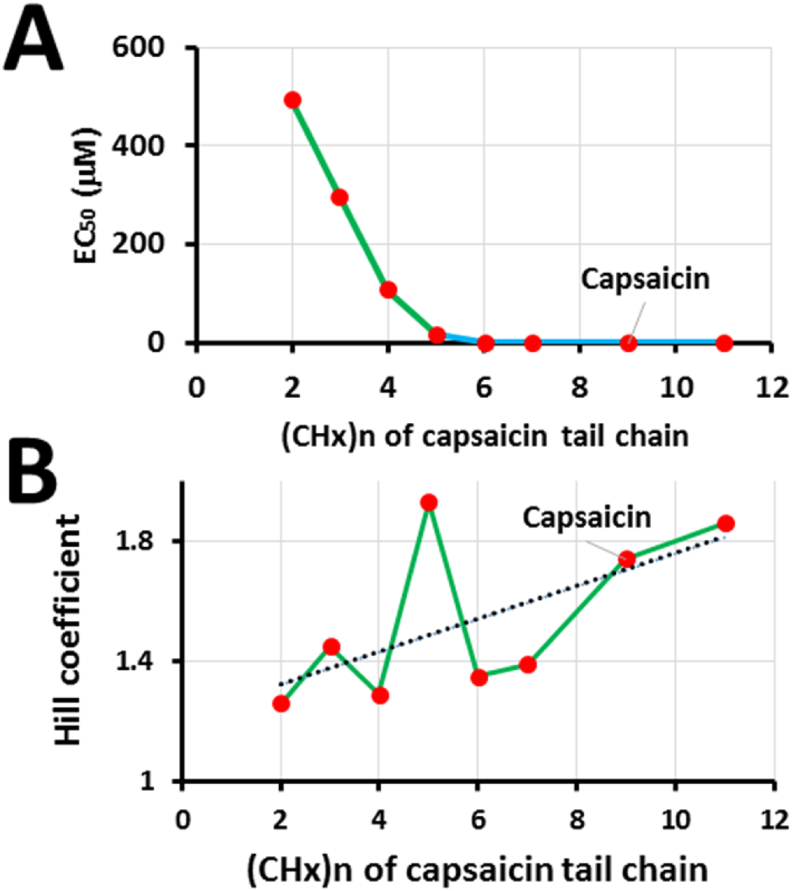
**The tail length-dependent changes in the Hill coefficient and the potency (EC**_**50**_**) of a capsaicin dose response for mTRPV1**. A long hydrophobic and flexible carbon chain serves as a tail of capsaicin. Both the capsaicin potency and the Hill coefficient increase with the increased tail length but have a sharp alteration when the n is five in (CHx)n. The long tail favors the swapping vanilloid bridge via the side chains between the anchor T671 on S6 and F544 on S4. Data were generated from the published article [5].

**Fig. 6:**
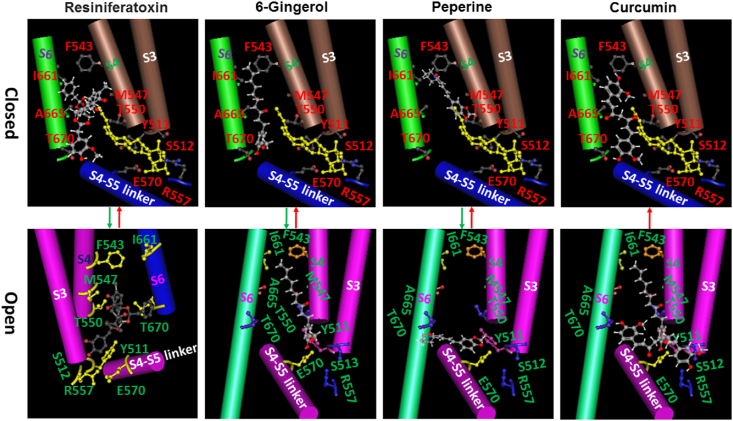
**The tentative lipid-dependent and anchor-stereoselective interactions between mTRPV1 and vanilloid compounds favor channel opening in a sequential cooperative mechanism**. The *in silico* protomer models were based on the cryo-EM structures of the rTRPV1 channel with a resident phosphatidylinositol lipid bound (PDB ID: 5IRZ) for the closed state or with capsaicin (PDB ID: 3J5R) or RTX (PDB ID: 5IRX) bound for the open state [3,4]. For convenience, only one vanilloid pocket is shown. S3, S4 and the S4–S5 linker are from one subunit while S6 is from the other neighboring subunit. The different rotamers of the residues in the putative binding pocket of TRPV1 were tested to optimally interact with the introduced agonists or antagonists in Fig. 2. The different vanilloid bridges between two separated active residues in Fig. 3 were also examined by the test molecules to make sure their spatial hindrance in the pocket to be minimal but their non-covalent interactions with nearby residues in the pocket to be maximal. In the primary closed state, the inositol ring of a phosphatidylinositol (PI) lipid (yellow) is anchored against S3 and the elbow of the S4–S5 linker, and further stabilized by polar interactions of the side chain of R557 on S4 with the OH- group of the phosphate on position 1, or of the side chain of E570 in the S4–S5 linker with a OH- group on position 6 of the inositol ring, and other electrostatic interactions (not shown). Lipid-free T670 on S6 may anchor the vanillyl head of RTX or 6-Gingerol/capsaicin while lipid-free T550 on S4 may act as an anchor for the vanillyl head of peperine to bind against. In this way, their long tail can be sandwiched via the inter-subunit side chains between F543 on S4 and I661 on S6 so that a recessive silent transient swapping vanilloid bridge can be formed to compete off the resident lipid. In contrast, curcumin has another large vanillyl group and thus cannot form the same bridge as 6-Gingerol does to compete off the resident lipid. When the resident lipid is released, a dominant steady-state stimulatory vanilloid bridge may be established via the intra-subunit side chains between the anchor T550 on S4 and S512 on S3 for RTX or E570 on the S4–S5 linker for 6-Gingerol/capsaicin. In that way, the reorientated Y511 ring may form a face-to-edge π−π interaction for the vanillyl ring to close the binding pocket so that the S4–S5 linker may be uplifted away from the S6 gate for channel opening. In contrast, the swapping peperine bridge between the hydroxyl group of Y511 on the S2–S3 linker and the anchor T670 on S6 may be exploited to uplift the S4–S5 linker away from the S6 gate for channel opening. In the open state, a silent swapping curcumin bridge via the side chains between the anchor T670 on S6 and S512 on S3 or R557 on S4 may compete off capsaicin or 6-Gingerol to inhibit TRPV1 opening. (For interpretation of the references to color in this figure legend, the reader is referred to the Web version of this article.)

The cryo-EM structures of rTRPV1 with bound vanilloid agonists showed that the side chains of F543 on S4 and I661 or A665 or T670 on S6 are lipid-free (Fig. 3, Fig. 6) [4]. However, their mutation in mTRPV1 almost doubly decreases the Hill coefficient of a capsaicin dose response but not dramatically change the capsaicin potency (Fig. 3, Fig. 4, Table 1) [5]. On the other hand, the Hill coefficient of a capsaicin dose response increases with the increased tail length of capsaicin (Fig. 5). Therefore, the long tail of capsaicin may be first sandwiched via the side chains between I662 on S6 from one subunit and F544 on S4 from the other neighboring one. The initial vanillyl group may H-bond with the side chain of T671 against S6 to compete off the resident lipid so that the vanilloid can bind to TRPV1 for allosteric activation (Fig. 6). Although this swapping vanilloid bridge is silent, this ligand binding against an anchor T671 on S6 in the lipid pocket may facilitate the release of the resident lipid and the formation of the stimulatory vanilloid bridge via the side chains between E570 on the S4–S5 linker and T550 against S4 (Fig. 3, Fig. 6). Thus, allosteric activation of TRPV1 by capsaicin is anchor-stereoselective. Since the vanilloid potency (EC_50_) is about 10 μM when the T551A/E571A mutation disrupts the second stimulatory vanilloid bridge via the side chains between T551 on S4 and E571 on the S4–S5 linker of mTRPV1, the initial vanilloid bridge via the side chains between T671 on S6 and F544 on S4 may be weak. On the other hand, this initial recessive transient silent ligand binding may promote the subsequent dominant steady-state active ligand binding and thus may favor the release of the occluded resident lipid and allosteric activation of TRPV1. Therefore, a sequential cooperative mechanism in which site accessibility controls cooperativity is reasonable. In this case, the high Hill coefficient of a dose response may be indicative of the concerted reaction steps or intermediates for allosteric activation of TRPV1 by capsaicin. Given that other vanilloid compounds or analogs also activate TRPV1 with a high Hill coefficient, it is necessary to examine which anchor favors each vanilloid agonist or analog to compete off the resident lipid and how many reaction steps or intermediates are needed to finally open allosteric TRPV1 channels.

### 7.1 The T671 anchor on S6 favors two-step activation of TRPV1 by capsaicin and resiniferatoxin and 6-Gingerol with a long tail

Since capsaicin and RTX and 6-Gingerol have a long tail and their dose response for activation of TRPV1 exhibits a high Hill coefficient of 2 (Table 1), these kinds of vanilloid agonists may target the anchor T671 on S6 to compete off the resident lipid after their tail is sandwiched via swapping N-terminals between S4 from one subunit and S6 from the other neighboring subunit.

Capsaicin (C_18_H_27_NO_3_, *trans*-8-Methyl-N-vanillyl-6-nonenamide) is a capsaicinoid. It has a vanillyl group as a head, an amide bond group as a neck and a long hydrophobic and flexible carbon chain as a tail. It has two H-bond donors and three H-bond acceptors but has not any defined atom stereocenter (Fig. 2). Its high lipid partition coefficient logP of 3.04 causes it to be insoluble in cold water but easily soluble in alcohol, ether and benzene. This vanilloid compound is colored in dark red and highly volatile with a pungent odor and tastes burning. Given that the Hill coefficient of a capsaicin dose response increases with an increased tail length (Fig. 5), the long tail of capsaicin may be first sandwiched via the side chains between I661 on S6 from one subunit and F543 on S4 from the other adjacent subunit. In the meantime the vanillyl group may H-bond with the anchor T670 against S6 of rTRPV1 (Fig. 6). Once capsaicin releases the resident lipid, its vanillyl group may further H-bond with the side chain of E570 on the S4–S5 linker while the amide group may H-bond with T550 on S4 of rTRPV1. In this way, the stimulatory vanilloid bridge between E570 and T550 can be formed together with extensive hydrophobic interactions between the aliphatic tail and residues lining the binding pocket for channel opening (Fig. 6).

6-Gingerol (C17H26O4, (5S)-5-hydroxy-1-(4-hydroxy-3-methoxyphenyl)-3-decanone) is a beta-hydroxy ketone and a member of guaiacols. Its LogP is 2.5. It has two H-bond donors and four H-bond acceptors and one defined atom stereocenter. 6-Gingerol and capsaicin have a similar neck or tail (Fig. 2). Once its vanillyl head is anchored against the side chain of T670 on S6, its tail is easily clamped via the side chains between I661 on S6 and F543 on S4 to compete off the resident lipid for activation of rTRPV1. After the resident lipid is released, its vanillyl group may subsequently H-bond with the side chain of E570 to pull the S4–S5 linker upward against the H-bond of its β-ketone group with the side chain of T550 on S4, promoting allosteric activation of rTRPV1 (Fig. 6).

Resiniferatoxin (RTX) (C_37_H_40_O_9_, 4-hydroxy-3-methoxy-benzeneacetic acid, [(2S,3aR,3bS,6aR,9aR,9bR,10R,11aR)-3a,3b,6,6a,9a, 10, 11, 11a-octahydro-6a-hydroxy-8,10-dimethyl-11a-(1-methylethenyl)-7-oxo-2-(phenylmethyl)-7H-2,9b-epoxyazuleno [5,4-e]-1,3-benzodioxol-5-yl]methyl ester) is the most potent functional analog of capsaicin. RTX is a special vanilloid complex with a large Y-shaped and hydrophilic tail. Its LogP is 4.5. It has two H-bond donors and nine H-bond acceptors and eight defined atom stereocenter (Fig. 2). Once the vanillyl group H-bonds with the side chain of T670 against S6, its large octahydro moiety is close to the side chain of M547 while the phenylmethyl group contacts with the side chain of A665 on S6 of rTRPV1. In these ways, when the resident lipid is released, the hydroxyl group of the vanillyl head may further H-bond with the side chain of S512 on S3 while its vanillyl aromatic ring facilitates a face-to-edge π-π interaction with the Y511 ring on the S2–S3 linker, and Y511 also favors a H-bond with the ester oxygen of RTX upon reorientation. The five-membered diterpene ring component of RTX is also stabilized by hydrophobic interactions with those amino acids such as L515, V518, M547, and I573 from one subunit, and L669 from a neighboring subunit. Once the carbonyl oxygen of its large octahydro moiety finally H-bonds with the side chain of the T550 anchor against S4, the stimulatory vanilloid bridge via the side chains between S512 on S3 and T550 on S4 drives the side chain of R557 to form a stimulatory salt bridge with the side chain of E570. In these ways, the S4–S5 linker is uplifted away from the S6 bundle crossing gate for the activation of rTRPV1 (Fig. 6) [4]. That is why RTX potency is very strong [2].

### 7.2 The T671 anchor on S6 also favors two-step inactivation of TRPV1 activity by capsazepine

Capsazepine (C_19_H_21_ClN_2_O_2_S, N-[2-(4-Chlorophenyl)ethyl]-1,3,4,5-tetrahydro-7,8-dihydroxy-2H-2-benzazepine-2-carbothioamide) is a synthetic analog of capsaicin. It belongs to catechols or thioureas or benzazepine or monochlorobenzenes. Its LogP is 3.9. It has three H-bond donors and three H-bond acceptors but has not any defined atom stereocenter. It has a hydroxylphenol group as a head and a large neck and a special tail ending in an aromatic chlorophenyl group (Fig. 2). The cryo-EM structure indicated that capsazepine shares the same binding site with capsaicin and RTX [4]. Because its dose response for suppression of capsaicin-evoked TRPV1 currents displays a higher Hill coefficient of 1.5 (Table 1) [5], once its chlorophenyl tail is sandwiched via the side chains between I661 on S6 and F543 on S4 of rTRPV1 in a swapping fashion, this antagonist may use its hydroxylphenol group to H-bond with the side chain of the T670 anchor on S6 to compete off capsaicin (Fig. 6). On the other hand, in the absence of a face-to edge π−π interaction with the Y511 ring or the steric bulk of the phenyl ring in the active site, although capsazepine is found to be orientated against S4 of rTRPV1 with the dihydroxy group H-bonding with the side chains of E570 on the S4–S5 linker and R557 on S4, the carbothioamide group cannot H-bond with the side chain of T551 to uplift the S4–S5 linker away from the S6 bundle crossing gate to stabilize the open state [4]. Accordingly, it factually inactivates TRPV1 with the higher Hill coefficient [5].

### 7.3 The T671 anchor on S6 also facilitates two-step activation of TRPV1 by peperine with a long neck

Peperine (C_17_H_19_NO_3_, 1-[(2E, 4E)-5-(1,3-benzodioxol-5-yl)penta-2,4-dienoyl]piperidine) is a N-acylpiperidine with piperidine substituted by a (1E, 3E)-1-(1,3-benzodioxol-5-yl)-5-oxopenta-1,3-dien-5-yl group at the nitrogen atom. It derives from an (E, E)-piperic acid. It is an alkaloid isolated from the plant *Piper nigrum*. It is a member of benzodioxoles, an N-acylpiperidine, a piperidine alkaloid and a tertiary carboxamide. Peperine is factually colourless white crystals but has aroma reminiscent of pepper. It can function as a NF-kappaB inhibitor, a plant metabolite, a food component and a human blood serum metabolite.

Peperine's LogP is 3.5 so that it is very slightly soluble in water but soluble in ether or oils. It has a benzodioxol group as a head and a penta-2, 4-dienoyl group as a neck but without a tail. It has 3 H-bond donors and 3 H-bond acceptors and has not any defined atom stereocenter but 2 defined bond stereocenters (Fig. 2). Given that activation of mTRPV1 by peperine exhibits a high Hill coefficient (h = 2.2) by targeting the capsaicin site, and the T551V mutation decreased the Hill coefficient from 2.2 to 0.9 (Table 1) [24], its benzodioxol head may H-bond with the T551 (T550 in rTRPV1) anchor on S4 for its neck to be sandwiched via the inter-subunit side chains between I662 (I661 in rTRPV1) on S6 and F544 (F543 in rTRPV1) on S4 (Fig. 3, Fig. 6). When the resident lipid is released, its dienoyl group may further H-bond with the side chain of T671 (T670 in rTRPV1) on S6 while its benzodioxol head may H-bond with the hydroxyl group of Y512 (Y511 in rTRPV1) to simulate TRPV1 opening (Fig. 6). This bridge may be weak and thus the efficacy is low [24].

### 7.4 The T551 anchor on S4 promotes one step activation of mTRPV1 by 6-Zingerone with a short tail

6-Zingerone (C_11_H_14_O_3_, 4-(4-Hydroxy-3-methoxyphenyl)butan-2-one) is the major pungent component in fresh ginger. It has yellowish to yellow-brown crystalline mass. It has sweet, spicy, warm, heavy floral, mildly animal balsamic, vanilla like odor. It is a member of phenols, a ketone and a monomethoxybenzene. It has one H-bond donor and three H-bond acceptors but has not any defined atom stereocenter (Fig. 2). Its LogP is 0.8 so that it is slightly soluble in water but very soluble in ethyl ether.

Activation of mTRPV1 by 6-Zingerone with a short tail has a low Hill coefficient of a dose response. However, the T551V mutation on S4 or the E570A mutation on the S4–S5 linker or the T671S mutation on S6 decreases its potency (Table 1) [25]. Therefore, 6-Zingerone may directly employs the stimulatory vanilloid bridge via the side chains between T551 on S4 and E571 on the S4–S5 linker or T671 on S6 to compete off the resident lipid for TRPV1 opening.

6-Shogaol (C17H24O3, (E)-1-(4-hydroxy-3-methoxyphenyl)dec-4-en-3-one) is a monomethoxybenzene, a member of phenols and an enone. Its LogP is 3.7. It has one H-bond donor and three H-bond acceptors and one defined atom stereocenter (Fig. 2). As 6-Shogaol also has a low Hill coefficient of a dose response (Table 1) [25], it is likely that the bent scorpion-shaped conformation may prevent the long tail from sandwiching via the inter-subunit side chains between F544 on S4 and I662 on S6 (Fig. 2). In this case, this vanilloid compound may stimulate mTRPV1 opening in the same manner as 6-Zingerone does.

### 7.5 S512 on S3 serves as an anchor for niacin to compete off the resident lipid for 3-step allosteric activation of hTRPV1

Niacin, also known as nicotinic acid or vitamin B3, is soluble in water. It is an essential B vitamin. When given in high doses, it is effective in decreasing low density lipoprotein (LDL) cholesterol and increasing high density lipoprotein (HDL) cholesterol. Therefore, it has a unique value in the therapy of dyslipidemia. On the other hand, niacin can cause mild-to-moderate serum amino transferase elevations. Its high doses and certain formulations have been related to clinically apparent, acute liver injury which can be severe as well.

When the hydrogen at position 3 of pyridine is replaced by a carboxy group, niacin becomes a pyridinemonocarboxylic acid or a pyridine alkaloid or a conjugate acid of a nicotinate. It can service as an antidote, an antilipemic drug, a vasodilator agent, a metabolite, a B vitamin, a nicotinamidase inhibitor, an *Escherichia* coli metabolite and a mouse metabolite.

Niacin is an odorless white crystalline powder with a feebly acid taste. It has a pKa of 4.75 and a LogP of 0.4 so that it is soluble in water. It has one H-bond donor and three H-bond acceptors but has not any defined bond stereocenter (Fig. 2).

**Fig. 7:**
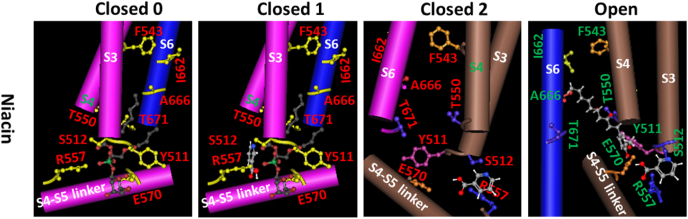
**The tentative lipid-dependent and anchor-stereoselective cooperative interactions between hTRPV1 and niacin favor channel opening in a non-swapping manner and the open state can be further stabilized by capsaicin or 6-Gingerol**. The *in silico* protomer models were based on the cryo-electron microscopy structural data of the rTRPV1 channel with lipid bound (PDB ID: 5IRZ or 3JSP) for the closed state or with capsaicin bound (PDB ID: 3J5R) for the open state [3,4]. For convenience, only one lipid pocket is shown. S3, S4 and the S4–S5 linker are from one subunit while S6 is from another neighboring subunit. The different rotamers of the residues in the putative binding pocket of TRPV1 were tested to optimally interact with the introduced agonists in Fig. 2. The different vanilloid bridges between two separated active residues in Fig. 3 were also examined by the test molecules to make sure their spatial hindrance in the pocket to be minimal but their non-covalent interactions with nearby residues in the pocket to be maximal. In the primary closed states 0 and 1, the inositol ring of a phosphatidylinositol (PI) lipid is anchored against S3 and the elbow of the S4–S5 linker, and further stabilized by polar interactions of the side chain of R557 on S4 with the OH- group of the phosphate on position 1, or of the side chain of E570 in the S4–S5 linker with a OH- group on position 6 of the inositol ring, and other electrostatic interactions (not shown). When the carboxyl group of niacin H-bonds with the side chain of R557 on S4 and its ring nitrogen H-bonds with the side chain of S512 on S3, the endogenous lipid is released but the hTRPV1 channel is still closed in state 2. Furthermore, the carboxyl group of niacin H-bonds with both the side chains of R557 on S4 and S512 on S3, its ring nitrogen H-bonds with the side chain of E570 on the S4–S5 linker, and its aromatic ring forms a face-to-edge π-π interaction with the Y511 ring upon reorientation to uplift the S4–S5 linker away from the S6 bundle crossing gate for channel opening. All these events are sequential and cooperative. Once capsaicin or its analog 6-Gingerol is applied, the vanillyl group may further H-bond with the side chain of E570 on the S4–S5 linker and the amide or ketone group may further H-bond with the side chain of T550 against S4. In that way, the open state can be further stabilized or enhanced by the stimulatory vanilloid bridge via the side chains between the anchor T550 on S4 and E570 on the S4–S5 linker in a cooperative manner.

Given that niacin activates hTRPV1 with a high Hill coefficient (h = 2.87) of a dose response (Table 1) [26], niacin may compete off the endogenous lipid by using the carboxyl group to H-bond with the side chain of R557 on S4 and the nitrogen atom of the pyridine head to H-bond with the side chain of S512 on S3 (Fig. 7). After the salt bridge between the side chain of R557 and the phosphate group of the resident lipid is disrupted by niacin, the resident lipid is released from TRPV1. Furthermore, the carboxyl group may H-bond with the side chain of E570 on the S4–S5 linker once the niacin ring forms a face-to-edge π-π interaction with the reoriented Y511 ring. In these ways, the S4–S5 linker is uplifted away from the S6 bundle crossing gate enough to open hTRPV1 channel.

When capsaicin or its analog 6-Gingerol is applied, the side chain of E570 can further H-bond with the vanillyl group to promote the H-bond formation between the amide or β-ketone group and the side chain of T550 against S4. Therefore, the stimulatory cooperative upward reorientation of the S4–S5 linker is further enhanced as shown in their stronger potency (Fig. 7). That's why both niacin and capsaicin can activate TRPV1 synergistically [26]. Given that niacin also decreases the threshold for heat activation of hTRPV1 from 38.92 °C to 24.88 °C [26], the salt bridge between the side chains of E570 and R557 may be favorable for activation of TRPV1 by heat [4]. On the other hand, although R557 may not contribute to the gating charge, the R558L mutation-induced decrease in V_1/2_ of mTRPV1 from 114.9 mV to 71.4 mV may be due to the release of the resident lipid for allosteric activation of TRPV1 upon membrane depolarization [27].

When capsazepine is applied, although its dihydroxy group can H-bond with the side chain of E570, the carbothioamide group cannot H-bond with the side chain of T550 against S4 to enhance the stimulatory uplift of the S4–S5 linker for channel opening. On the other hand, the H-bonding of the dihydroxy group with the side chain of E570 slightly weakens the H-bond between the side chain of E570 and the ring nitrogen of the pyridine head. In this way, capsazepine can slightly inhibit niacin-evoked activation of TRPV1 (Fig. 7).

### 7.6 Curcumin cannot use the anchor T671 on S6 to release the resident lipid but can exploit the anchor S512 on S3 to compete off capsaicin from the stimulatory binding site

Curcumin is a bright yellow natural phytopolylphenol from the root of *Curcuma longa* or turmeric which is a member of the ginger family, *Zingiberaceae*. It has a variety of pharmacologic properties. For example, it can serve as a metabolite, an anti-inflammatory agent, an antineoplastic agent, a hepato-protective agent, a flavouring agent, a biological pigment, a nutraceutical, an antifungal agent, a dye, a lipoxygenase inhibitor, a ligand, a radical scavenger, a contraceptive drug, a histone deacetylase inhibitor, an immunomodulator, an iron chelator, a neuroprotective agent, a herbal supplement, a cosmetics ingredient, a food colouring, an aldehyde reductase inhibitor, a shikimate dehydrogenase inhibitor, a IMP dehydrogenase inhibitor, an [NAD(P)H dehydrogenase (quinone)] inhibitor, an thioredoxin reductase inhibitor and a non-specific protein-tyrosine kinase inhibitor.

Chemically, curcumin is a diarylheptanoid, belonging to the group of yellow curcuminoids. It derives from a hydroxycinnamic acid. Since it incorporates two α, β-unsaturated carbonyl groups with two symmetric vanillyl (O-methoxy-phenolic) groups, it is a tautomeric compound. The diketones form stable enols in water and are readily deprotonated to form enolates in organic solvents. Its LogP is 3.29 and thus it is poorly soluble in water but very soluble in ethanol or acetic acid. It has two H-bond donors and six H-bond acceptors but has not any defined atom stereocenter (Fig. 2).

Curcumin or capsazepine has been reported to inhibit the histamine- or capsaicin-evoked inward currents of mTRPV1 in capsaicin-sensitive mouse DRG neurons. The resultant dose response exhibits a Hill coefficient as low as 0.9 (Fig. 2, Table 1) [28]. However, curcumin has no effect on membrane currents from *Xenopus* oocytes expressing rTRPV1 [29]. In this regard, curcumin may not compete off the resident lipid as a result of the large tail and neck even if its vanillyl head can H-bond with the side chain of T670 against S6 (Fig. 2). On the other hand, once capsaicin has activated TRPV1, curcumin can compete off capsaicin by using one vanillyl head to H-bond with the side chain of T670 again S6 and the other vanillyl tail to H-bond with the side chains of S512 on S3 and R557 on S4. Since this swapping vanillloid bridge between S512 and T670 may be silent, the capsaicin- or histamine-evoked TRPV1 current is suppressed (Fig. 6) [28].

It is interesting that curcumin cannot inhibit the heat-activated TRPV1 current [30]. Thus, if heat releases the resident lipid to form the stimulatory salt bridge between E570 on the S4–S5 linker and R557 on S4 as previously proposed [4], this silent swapping curcumin bridge between S512 on S3 and T670 on S6 may not affect the stimulatory E570-R557 salt bridge.

Taken together, all these vanilloid compounds or analogs except 6-Zingerone have a scorpion-shaped bend conformation in the solid state. However, they have an extended conformation in solution. Their hydrophilic head and neck can act as H-bond donors or acceptors. In contrast, their hydrophobic tails contain aliphatic carbon chains with a variety of lengths and sizes. These different structural properties may account for their diversity as an agonist or an antagonist of TRPV1. Except for curcumin, other vanilloid compounds or analogs can specifically bind to a lipid-free anchor to release the resident lipid for the modulation of the functional properties of TRPV1 in a sequential cooperative fashion. Given that both capsaicin and its analog curcumin can potentiate CFTR activity but with high and low Hill coefficients of a dose response, respectively [[31], [32], [33], [34]], it is promising to determine if there exist several cooperative capsaicin sites or multi-step reaction intermediates for allosteric activation of CFTR by capsaicin [31].

## 8 Membrane hyperpolarization may stabilize the resident lipid while membrane depolarization may facilitate the release of the resident lipid regarding allosteric activation of TRPV1

A phosphatidylinositol lipid has a polar head group and a non-polar tail and thus serves as an amphiphile. It belongs to a glycerophospholipid once including a glycerol backbone, two non-polar fatty acid tails and a phosphate group substituted with an inositol polar head group. The phosphate group gives the molecule a negative charge at physiological pH (Fig. 2). Given that the heat efficacy has doubled the capsaicin or RTX efficacy regarding allosteric activation of rTRPV1 at membrane hyperpolarization but this difference in efficacy disappears at membrane depolarization [2,27,35], it is more likely than not that membrane hyperpolarization may facilitate the resident lipid binding to the cytoplasmic inter-subunit interface between the VSLD and the pore domain. In contrast, membrane depolarization may promote the release of the resident from the cytoplasmic side (Fig. 5, Fig. 7). Thus, membrane depolarization can increase the vanilloid efficacy by easily removing the resident lipid. On the other hand, membrane depolarization may decrease the Hill coefficient possibly by reducing the sequential reaction intermediates or steps for allosteric activation of TRPV1. That's why the Hill coefficients of a dose response for capsaicin, 6-Shogaol, 6-Gingerol or 6-Zingerone from calcium imaging results are higher than those from electrophysiological measurement results [25].

## 9 Closing remarks

In summary, dominant steady active ligand binding sites in biological macromolecular receptors including voltage- and ligand-gated ion channels have been extensively revealed by high throughout random scanning, or site-directed mutagenesis studies, or chimera comparison analysis between a ligand-sensitive species and a ligand-insensitive one, or X-ray crystal or cryo-EM structural determination. However, the information about the recessive transient silent ligand binding sites, though important, is still less known, partly because a current structural tool or method has not got those sites. In this comparative *in silico* research, cross-examination of changes in the ligand potency and the cooperative dose response regarding the allosteric activation of TRPV1 by a variety of vanilloid compounds and analogs may provide an important method or resource to uncover those recessive transient silent ligand binding sites. If the Hill coefficient of a ligand dose response changes with the varying ligand potency, an inter-subunit sequential cooperative mechanism may be applied. However, if the Hill coefficient changes without the altering ligand potency, an intra-subunit but inter-site sequential cooperative mechanism may be taken, and there may exist at least one recessive transient silent ligand binding intermediate for an allosteric biological macromolecular receptor. In this regard, this review may enhance our understanding of regulation mechanisms of an allosteric biological macromolecular receptor.

Unlike the ligand-stereoselective allosteric activation of cold-sensing TRPM8 channels by an H-bonded homochiral menthol dimer with head-to-head or head-to-tail [37], an unusual feature of the significant allosteric activation of TRPV1 by a vaniloid compound or analog is that the resident and occluded lipid must be competed off. A lipid-free anchor clearly facilitates the ligand binding to remove the resident lipid from the active site. Furthermore, the anchor stereoselectivity depends on if the ligand tail or neck can be stabilized by residues at the active site upon non-valent interactions to form a putative vanilloid bridge with the anchor. Different anchor stereoselectivities can produce diverse recessive transient reaction intermediates or steps for allosteric activation of TRPV1 in a sequential cooperative mechanism. In that regard, this lipid-dependent anchor stereoselectivity for sequential allosteric activation of TRPV1 by vanilloid compounds or analogs may have significant and extensive and mechanistic implications for other lipid-dependent allosteric membrane transporters.

In the future, the active site-directed mutation, together with the structural determination and the measurement of the site- and potency-dependence of the Hill coefficient of a ligand dose response, may provide more valuable clues about those recessive transient silent ligand binding intermediates and their roles in the stereoselective interplay between a ligand and an allosteric biological macromolecular receptor.

It should be noteworthy that although the vanilloid pocket is located in the transmembrane domains, binding of phospholipids such as the signaling lipid phosphoinositide4,5-bisphosphate (PI(4,5)P_2_) or its precursor phosphoinositol 4-phosphate (PI_4_P) on the N- and C-terminal domains can act as cofactors or allosteric inhibitors or both to modify the vanilloid sensitivity of TRPV1. Their depletion by Ca^2+^ -induced activation of phospholipase Cδ isoforms (PLCδ) limits channel activity during capsaicin-induced desensitization [[38], [39], [40]]. Accordingly, care must be taken for the *in vivo* studies when the Hill coefficients and/or the potency of the vanilloid ligands may be determined not only by the interaction between the vanilloid ligands and their binding domain but also the modification of the N- and C-terminal domains by those phospholipids.

## Declaration of competing interest

The author declares no conflict of interest.

## Abbreviations

BPRC: bacterial photosynthetic reaction center
CFTR: cystic fibrosis transmembrane conductance regulator
CNG: cyclic-nucleotide-gated
cGMP: cyclic guanosine monophosphate
CTQ: cysteine tryptophylquinone
EM: electron microscopy
DkTx: double-knot toxin
DRG: dorsal root ganglia
HDL: high density lipoprotein
ICK: inhibitor cysteine knot
LDL: low density lipoprotein
PBRC: purple bacterial reaction center
PlGoxA: *Pseudoalteromonas luteoviolacea*
PI(4,5)P_2_: phosphatidylinositol-4,5-bisphosphate
PI(4)P: phosphoinositol 4-phosphate
PLCδ: phospholipase Cδ isoforms
RTX: resiniferatoxin
TG: trigeminal ganglia
TRP: transient receptor potential
TRPA1: TRP ankyrin-1
TRPM8: transient receptor potential melastatin 8
TRPV1: TRP vanilloid-1
hTRPV1: human TRPV1
rTRPV1: rat TRPV1
mTRPV1: mouse TRPV1
gTRPV1: chicken TRPV1
oTRPV1: rabbit TRPV1
hTRPV2: human TRPV2
mTRPV2: mouse TRPV2
hTRPV3: human TRPV3
hTRPV4: human TRPV4
hTRPV5: human TRPV5
hTRPV6: human TRPV6
VSLD: voltage sensor-like domain
